# Production and Characterization of Bacterial Ghost Vaccine against *Neisseria meningitidis*

**DOI:** 10.3390/vaccines11010037

**Published:** 2022-12-23

**Authors:** Randa H. Ali, Mohamed E. Ali, Reham Samir

**Affiliations:** 1 Department of Microbiology and Immunology, National Organization for Research and Control of Biological (NORCB), Giza 12622, Egypt; 2 Department of Microbiology and Immunology, Faculty of Pharmacy, Cairo University, Cairo 11562, Egypt

**Keywords:** *Neisseria meningitidis*, ghost vaccine, polysaccharide vaccine

## Abstract

Bacterial ghosts (BGS) are empty non-living envelopes produced either genetically or chemically. This study investigated a novel chemical protocol for the production of *Neisseria meningitidis* ghost vaccine using tween 80 followed by a pH reduction with lactic acid. For our vaccine candidate, both safety and immunogenicity aspects were evaluated. The ghost pellets showed no sign of growth upon cultivation. BGS were visualized by scanning electron microscopy, illustrating the formation of trans-membrane tunnels with maintained cell morphology. Gel electrophoresis showed no distinctive bands of the cytoplasmic proteins and DNA, assuring the formation of ghost cells. In animal model, humoral immune response significantly increased when compared to commercial vaccine (*p* < 0.01). Moreover, serum bactericidal assay (SBA) recorded 94.67% inhibition compared to 64% only for the commercial vaccine after three vaccination doses. In conclusion, this is the first *N. meningitidis* ghost vaccine candidate, proven to be effective, economic, and with significant humoral response and efficient SBA values; however, clinical studies should be performed.

## 1. Introduction

Vaccination is the cornerstone of protecting humans from pathogens. Traditional vaccines, either inactivated or live attenuated, usually resemble the disease causative agent but in a beneficial way. They stimulate the immune system to produce humoral and/or cellular immunity [1]. A novel approach to vaccine production is bacterial ghost (BG) preparation, which is a killed empty cell envelope with a conserved cell wall. BG can stimulate both innate and acquired immunity by the displayed antigens on its intact cell wall. Moreover, it can be used as an adjuvant or be filled with drugs to be delivered to the site of infection [2]. BG is prepared by several methods, which is the controlled expression of the cloned lysis gene *E* technique, which is only suitable for Gram-negative bacteria ghost cell production [3]. However, this method is highly complicated and expensive. Besides, it may need the addition of some enzymes or chemicals to achieve complete inactivation [2]. On the other hand, the chemical method for BG preparation is based on the determination of the minimal inhibitory concentrations of certain chemicals, for example, NaOH [4]. This method has the advantage of being feasible and easy to apply. Moreover, it can be used for both Gram-negative and Gram-positive bacterial ghost production [4]. Unfortunately, NaOH has a considerable damaging effect on the antigens present on the cell wall, leading to a partial loss of ghost cell antigenicity and immunogenicity [5,6]. As an attempt to decrease this damaging effect on the cell wall antigens, another protocol published in 2018 suggested exposing the bacteria to low concentrations of tween 80 followed by lactic acid addition to decrease the pH [6]. Notably, tween 80 had FDA approval to be used in food [7] and was proven to have a significant effect on slowing the growth rate and decreasing the biofilm formation of the Gram-negative bacteria *Pseudomonas fluorescens* [8]. In addition, the lactic acid concentration used in Rabea et al. protocol [6] facilitates the penetration of the cytoplasmic membrane of many Gram-negative bacteria, such as *Escherichia coli* O157:H7, *P. aeruginosa,* and *Salmonella enterica serovar Typhimurium.* It sensitizes the cell to the lytic action of lysozymes or certain chemicals as SDS [9].

Meningitis, if untreated, is a fetal disease of the meninges (membranes covering the brain and spinal cord). It can be caused by bacteria, fungi, or viruses, but the highest global percentage is attributed to bacterial meningitis. *Streptococcus pneumoniae*, *Haemophilus influenzae*, and *Neisseria meningitidis* are the leading meningitis causative agents [10]. *N. meningitidis* is the main cause of epidemic meningitis. It is Gram-negative oval cocci that may appear in pairs with adjacent sides flattened or concave. The *N. meningitidis* capsule is composed of polysaccharides [11].

Twelve serogroups of *N. meningitidis* have been identified, but only six of them (A, B, C, W, X, and Y) can cause epidemics. *N. meningitidis* can be transmitted through droplets of respiratory or throat secretions. African meningitis belt represents the largest percentage of this disease, where serogroup A causes approximately 80% of the cases [10]. By 2010, mass immunization with meningococcal A conjugate vaccine has started, and the incidence of the disease has approximately disappeared. Nowadays, the available vaccines are meningococcal A conjugate, C conjugate, tetravalent A, C, Y, and W conjugates, and meningococcal polysaccharide vaccines [12]. On the one hand, the purified polysaccharides (T cell-independent) have many disadvantages, for instance; their immunogenicity is age-related (not given below 2 years), they fail to elicit immune response upon several immunizations and fail to induce immunological memory at any age [11]. On the other hand, the conjugated vaccine is T cell-dependent that induces serum IgG antibodies and memory B cells [13]. However, it is not affordable to be included in the developing countries’ expanded program of immunization schedules.

Unfortunately, 17 outbreaks have been reported in nine different African countries (Benin, Burkina Faso, Chad, Côte d’Ivoire, Ghana, Mali, Niger, Nigeria, and Togo) from 2011 to 2017. Serogroup C was the predominant reason for 11 outbreaks, while the rest of them were caused by serogroup W. By 2018, the epidemic level decreased when compared to 2017; however, serotype C (NmC) continued to cause outbreaks in Nigeria and Niger. In 2017–2018, WHO and its partners called for increasing the availability of the global NmC vaccine in the African belt to control the epidemic activity, which remains largely unpredictable [14].

This unpredictable epidemic behavior and the well-known disadvantages of the currently in-use purified meningococcal polysaccharide vaccine [11] encourage us to study the production of *N. meningitidis* ghost serotype C vaccine (NmC-BG) by novel chemical lysis method using tween 80 [6]. This vaccine was evaluated in terms of safety and efficacy in animal model and compared to the pre-existing commercial meningococcal polysaccharide AC vaccine (walvax).

## 2. Materials and Methods

### 2.1. Bacterial Strain, Cultivation, and Storage Conditions

*N. meningitidis* serotype C (ATCC 13102) strain was purchased from Mecconti^TM^, Warsaw, Poland. It was the 2nd passage from the reference in the form of pellets. One pellet was reconstituted, spread on blood agar (Animal Health Research Institute, Giza, Egypt) under biological safety cabinet (Thermo Scientific, MS, USA), and incubated at 37 °C for 18–24 h in 5% CO_2_ incubator (Kendro laboratory products, Hanau, Germany) [15]. The colonies were harvested in brain heart infusion (BHI) broth (Merck KGaA, Darmstadt, Germany)/glycerol (Fisher, Winsford, U.K.) dispensed in sterile micro-centrifuge tubes and stored at −80 °C (Thermofisher, MS, USA). Whenever needed, a tube of *N. meningitidis* serotype C culture was prepared by colony suspension method: inoculation of harvested bacteria pellets on a BHI agar (Merck KGaA, Darmstadt, Germany) plate. The isolated colonies from an overnight culture were selected and added to a tube containing BHI broth (Merck KGaA, Darmstadt, Germany) [16].

### 2.2. Determination of the Minimum Inhibitory Concentration (MIC) of Lactic Acid

In order to determine the MIC, sterile tubes of 10 mL pre-filtered BHI broth solution containing 7% tween 80 (Fisher scientific, Winsford, U.K.) were used, in which 100 µL of adjusted culture (0.5 McFarland) were added to each tube and incubated at 37 °C for 24 h in 5% CO_2_ incubator. At the end of incubation period, lactic acid solution (Piochem, Giza, Egypt) was pre-sterilized using 0.45 µ filter syringe (Sartorius, Göttingen, Germany) then added to the tubes in different volumes starting from 100 µL to 1000 µL with final concentration of 1% to 10% [17]. The turbidity of each tube was assessed visually.

In addition, the tubes with no visible growth signs were verified by spreading 100 µL on BHI agar (Merck KGaA, Darmstadt, Germany) and incubated at 37 °C for 24 h in 5% CO_2_ incubator. The MIC value was determined from three independent experiments.

### 2.3. Production of NmC-BG

Production of NmC-BG was carried out using Rabea et al. protocol [6]. In brief, nine sterile tubes of 10 mL pre-filtered BHI broth solution containing 7% Tween 80 were inoculated with 100 µL of the adjusted culture of *N. meningitidis* serogroup C (0.5 McFarland) and incubated at 37 °C for 24 h in 5% CO_2_ incubator. This was followed by the addition of 200 µL of the pre-sterilized lactic acid pre-determined in Section 2.2; to reach a pH of 3.28. All the tubes were incubated at 37 °C for 1 h in a 5% CO_2_ incubator. The experiment was carried out in triplicates in which NmC-BG cells were harvested by centrifugation for 30 min at 4 °C, 4000 rpm (Eppendorf, Hamburg, Germany). The supernatant was stored in the refrigerator (Frimed, Turin, Italy) for the quantification of proteins and DNA. The pellets were washed with sterile half-normal saline solution twice and kept at 2–8 °C until use.

### 2.4. Identification Tests

#### 2.4.1. Detection of NmC-BG Formation by Scanning Electron Microscopy (SEM)

BG pellets and untreated bacterial cells were coated with gold, and the morphological analysis was performed by SEM (Quanta FEG 250, CA, USA) [6].

#### 2.4.2. Agglutination Test

Latex agglutination test gives rapid, sensitive, and serogroup-specific slide identification test of *N. meningitidis*. A Pastorex Meningitis kit (Bio-Rad, Paris, France) was used. On a clean glass slide, 10 µL of *N. meningitidis* C Latex R7 (red latex suspension sensitized with rabbit antibodies specific for *N. meningitidis* C) was added to BG pellets. A negative control was carried out by adding negative polyvalent control latex (R9) (latex suspension sensitized with IgG from non-immunized rabbits) to the BG pellets. Agglutination was observed macroscopically within a maximum of 10 min [18].

### 2.5. Analysis of Protein

#### 2.5.1. Quantification of Released Proteins

A Pierce BCA protein assay kit (Thermo-fisher, MS, USA), a detergent-compatible protein assay [19], was used to determine the quantity of protein (µg/mL) released in the broth during the production of BG using a Spectronic Unicam UV-Vis spectrophotometer (Helios alpha, Leeds, U.K.). The protein contents of un-inoculated media were measured and considered in the calculation to nullify the protein content of the cultivation media [6]. The experiment was carried out in triplicates, and the average was calculated.

#### 2.5.2. Protein Gel Electrophoresis and Western Immunoblotting Analysis

##### Protein Gel Electrophoresis

To analyze the remaining protein content in BG cells, SDS-PAGE was performed, in which NmC-BG and untreated bacterial cells were adjusted to 0.5 McFarland. In addition, supernatants obtained from both treated and untreated bacteria suspension after dilution (1/15) were denatured using reducing buffer (2 mL of 10%SDS (Serva, Heidelberg, Germany), 0.196 gm DTT (Merck KGaA, Darmstadt, Germany), 2 mL glycerol anhydrous (Fluka, Frankfurt, Germany), 2 mL of 0.5 M sodium phosphate monobasic monohydrate (Sigma-Adrich, MO, USA), 0.1 mL of 1% Bromophenol Blue (BioRad, Shanghai, China) and 3.8 mL of purified water). Twelve percent Mini-PROTEAN TGX precast protein gel (BioRad, CA, USA) was mounted in a gel electrophoresis device (BioRad, CA, USA) then all samples were loaded against a protein marker. Samples were run under a constant current of 100 V. The gel was stained in Coomassie brilliant blue solution (BioRad, Shanghai, China) and immersed in de-staining solution (45% methanol (Sigma, MO, USA); 10% acetic acid (Carlo Erba, Normandy, France); 45% distilled water) until the bands were visually detectable [20].

##### Western Immunoblotting Analysis

The antigenic protein in NmC-BG cell walls were investigated [21]. In brief, BG pellets were adjusted to 0.5 McFarland then denatured using reducing buffer as mentioned in Section 2.5.2. Twelve percent Mini-PROTEAN TGX precast protein gel (Bio-Rad, CA, USA) was mounted in gel electrophoresis device (BioRad, CA, USA) then samples and pre-stained protein marker were loaded. Samples were run under constant current of 100 V. After separation was completed, the gel and nitrocellulose membrane were immersed separately in transfer buffer (3.02 gm tris, 14.4 gm glycine, 800 mL purified water, 100 mL methanol, mixed well and completed to 1000 mL with purified water) for 30 min. Filter papers and sponges were also soaked in transfer buffer for 5 min. The transfer stack was assembled in the cassette holder and the cassette was mounted in the transfer tank, which was filled with transfer buffer. The system was turned on for 1 h at 100 V. After that, the stack was disassembled and the nitrocellulose membrane (BioRad, CA, USA) was immersed in blocking buffer (10 mL of fetal bovine serum (Thermo-fisher, MA, USA), 90 mL of TTBS buffer (12.1 gm of tris, 9 gm of sodium chloride (Advent, Mumbai, India), 1.1 mL of tween 80 and purified water adjusted at pH 7.5 with HCl (Thermo-fisher, MA, USA)) for 60 min. The solution was discarded and the membrane was immersed in 20 mL of TTBS buffer containing 0.5 mL rat vaccine derived antibodies and kept overnight at room temperature. The membrane was washed three times with TTBS buffer then rabbit anti-mouse IgG peroxidase conjugated (Sigma-Aldrich, MO, USA) diluted 1:8000 in TTBS buffer to a total volume of 20 mL was added on the membrane for 40 min at room temperature. The membrane was washed three times with TTBS buffer then the liquid was discarded. DAB substrate kit (Abcam, BOS, USA) was added on the membrane in the dark until the bands appeared.

### 2.6. Determination of DNA Content

The DNA released in the supernatant and the remaining genomic DNA inside the NmC-BG pellets were determined after extraction. Extraction of DNA was performed in two steps, precipitation of protein followed by precipitation of the DNA.

#### 2.6.1. Precipitation of Protein

For BG and untreated bacterial cells, the DNA was extracted from both BG and untreated bacterial pellets (adjusted to 0.5 McFarland), in which the pellets were separated from their culture by centrifugation for 30 min at 4 °C, 4000 rpm. In brief, 1 mL of preheated SDS lysis buffer [20 gm SDS/L (Serva, Heidelberg, Germany), 150 mM NaCl (Advent, Mumbai, India), 100 mM Tris/HCl (Techno pharmchem, New Delhi, India), 25 mM EDTA (Bio basic, ON, Canada), pH 8] was added to the pellets, mixed then 10μL Proteinase K (Serva, Heidelberg, Germany) was added and tubes were incubated at 65 °C for 1 h followed by centrifuging for 10 min at 12,000× *g* [22]. To precipitate the protein, 300 µL of 7.5 M stock solution of ammonium acetate (Advent, Mumbai, India) was added to 600 µL of supernatant collected after extraction, so the final concentration of ammonium acetate reached 2.5 M. This mixture was kept for 5 min on ice, vortexed for 5 s, then a clear supernatant containing DNA was collected by centrifugation at 12,000 rpm for 10 min at room temperature [23].

For the supernatant of BG culture: 600 µL was added to 300 µL of 7.5 M stock solution of ammonium acetate (Advent, Mumbai, India) to reach a final concentration of 2.5 M. This mixture was incubated for 5 min on ice, vortexed for 5 s followed by centrifugation at 12,000 rpm for 10 min at room temperature. The clear supernatant containing DNA was collected [23].

#### 2.6.2. Precipitation of DNA

Fifty µL of sodium acetate stock solution (3 M) (CDH Ltd., New Delhi, India) were added to 450 µL of the supernatant obtained after protein precipitation (from Section 2.6.1) so that the final concentration of sodium acetate reached 0.3 M. Ice-cold absolute ethanol (Chem-lab, Zedelgem, Belgium) was added to each solution and vortexed for 5 s, incubated for 4 h at −40 °C, and then the samples were centrifuged at 14,000× *g* for 1 h at 4 °C. The supernatant was discarded, and the pellet was washed twice with 75% ice-cold ethanol (Alfa chemical, Cairo, Egypt), in which each step was followed by centrifugation at 14,000× *g* for 10 min at 4 °C. A final wash with 100% ice-cold isopropanol (Serva, Heidelberg, Germany) was performed, then the wash solution was removed, and the pellet was left to air dry in the biological safety cabinet. After drying, 10 μL of 1X TE (Himedia, Mumbai, India) was added to the pellet, and it was incubated at 55 °C for 10 min for solubilization. The obtained DNA was stored at −80 °C until use [23].

Quantities of obtained DNA were determined using the NanoDrop 2000/2000c spectrophotometer (Thermo Scientific, MA, USA). The ratio of absorbance at 260 nm/280 nm was measured to evaluate DNA purity [6].

#### 2.6.3. DNA Gel Electrophoresis

The DNA extracted from untreated bacterial cells, as well as the BG pellets and the supernatant of BG culture, was examined. Electrophoresis was performed on all extracted DNA samples using 1% agarose gel (Sigma-Aldrich, MO, USA) containing ethidium bromide (Fisher, Winsford, U.K.) at constant 100 V for about 1 h. The gel was visualized under the UV gel documentation system (Maxlife, London, U.K.). DNA ladder 1 kbp (Serva, Heidelberg, Germany) was used [4].

### 2.7. Safety Assessment

#### 2.7.1. Viability Test

The prepared BG pellets were evaluated for the existence of any viable cells, where samples were spread on blood agar plates. The plates were then incubated at 37 °C in a 5% CO_2_ incubator for three days [4]. Plates were examined visually for the presence of any colonies.

#### 2.7.2. Abnormal Toxicity Test

This test was performed according to European Pharmacopoeia 8.0 monograph 01/2008: 20609 for human vaccines. Five Swiss albino mice weighing 17–22 g, in addition to 2 healthy guinea pigs weighing 250–350 g, were inoculated via intra-peritoneal (IP) route at a dose of 0.5 mL/animal (BG vaccine adjusted 0.5 McFarland; 10^8^ CFU/mL). All the inoculated animals were observed daily for any signs of illness during the 7-day observation period.

#### 2.7.3. Bacterial Endotoxin Test

This test is a substantial requirement for the safety of all injectable pharmaceutical products. Bacterial endotoxin is a type of pyrogenic substance found in the cell wall of Gram-negative bacteria and should be controlled, otherwise causing fever, septic shock, or death [24]. Therefore, we measured the quantity of endotoxin by using bacterial endotoxin USP monograph 85 (method: gel-clot, quantitative test). In brief, the NmC-BG suspension was adjusted to 0.5 McFarland (10^8^ CFU/mL) using LAL water under the biological safety cabinet (Thermo Scientific, MA, USA). Single-test LAL reagent sensitivity 0.25 EU/mL (Charles River, MA, USA) was used. Ten-fold dilution was performed of the BG vaccine, added on the reagent and then incubated at 37 ± 1 °C for 60 ± 2 min in a water bath (Memmert GmbH, Schwabach, Germany). The result was calculated according to the USP monograph. If none of the dilutions is positive (gel formation), the endotoxin concentration is reported as less than LAL reagent sensitivity multiplied by the lowest dilution factor.

### 2.8. Immunogenicity Assessment

#### 2.8.1. Induction of Antibodies Response

Thirty healthy male rats weighing 100 ± 10 g were purchased from the animal facility of Theodor Bilharz Research Institute (Giza, Egypt) and housed in the animal house of the Egyptian Drug Authority (EDA) (Agouza, Giza, Egypt). Animals were housed in a controlled environment at 22 ± 3 °C, 55 ± 5% humidity, and 12 h light/dark cycle. They were provided with a standard laboratory diet and water. The rats were adapted to their environment for one week before starting the experiment. All experimental work on animals was conducted according to international guidelines and approved by the Ethical Committee in the Faculty of Pharmacy, Cairo University (code: MIC-3.12.1).

The vaccines used in this experiment were the commercial meningococcal A&C (Walvax company) which contains 50 µg of each polysaccharide serogroups A and C per 0.5 mL dissolved in sterile PBS and our newly developed NmC-BG vaccine prepared by suspending the BG pellets in sterile PBS and adjusting it to 0.5 McFarland (10^8^ CFU/mL).

Experimental design: Five groups of rats (n = 6 rats/group) were immunized in which each rat received a single dose of 50 µg of A&C vaccine (Walvax) via intramuscular (IM) and subcutaneous (SC) routes for groups 1 and 2. While each rat received 0.5 mL of NmC-BG via IM and SC routes for groups 3 and 4, immunization was conducted on days 0, 14, and 28. On the other hand, in group 5, each rat received PBS only (negative control). Blood was collected via a retro-orbital route on days 14, 28, and 42 from the first injection, and sera were separated by centrifugation for 10 min at 2500 rpm. Separated sera were pooled per each group and stored at −20 °C until testing for antibodies responses against meningococcal C (IgG) by ELISA technique [25].

#### 2.8.2. Assessment of Immune Response in Rat Groups Sera

Sera antibody (IgG) levels elicited in rats’ groups were evaluated by indirect ELISA [25]. Briefly, ELISA plates (NUNC, NY, USA) were coated by adding 100 µL of adjusted 0.5 McFarland (10^8^ CFU/mL) in carbonate buffer and incubated at 2–8 °C overnight. Plates were washed three times with PBS (Bio basic, ON, Canada) containing 0.05% Tween 20 (Sigma, MO, USA) (PBS-T) and blocked with 200 µL per well of 1% bovine serum albumin (BSA) (Sigma, MO, USA) in PBS-T for 2 h at room temperature; then, plates were washed three times with PBS-T. Sera were serially diluted two-fold in PBS-T/1% BSA. The plates were incubated for 2 h at room temperature; after that, the plates were washed three times with PBS-T. A total of 100 µL rabbit anti-mouse IgG peroxidase (1:7000; Sigma–Aldrich, MO, USA) in PBS-T/1% BSA was added, and the plates were incubated for 2 h at room temperature. The plates were washed with PBS-T. A total of 100 µL of the chromogen substrate solution, TMB (Sigma, MO, USA), was added, and the plates were incubated for 20 min; then, 100 µL of stop solution 1N H_2_SO_4_ (Sigma–Aldrich, MO, USA) were finally added. Absorbance was measured at 450/620 nm by an ELISA reader (Tecan, Männedorf, Switzerland). The ELISA results were calculated in terms of the cut-off formula from “2 X mean of negative controls” [26].

#### 2.8.3. Serum Bactericidal Activity (SBA)

A total of 20 µL of sera was added to 60 µL of sterile PBS. A total of 20 µL of this mixture was added to 100 µL of bacterial suspension (1 × 10^6^ CFU/mL) and incubated at room temperature for 1 h. After incubation, 100 µL was spread on blood agar and then incubated at 37 °C for 24 h. A negative control was conducted in which the serum was replaced by sterile PBS. The number of colonies on the plates was counted, and the percentage of bactericidal activity was calculated using the formula: SBA = [1 − (the number of viable bacteria after serum treatment/the number of viable bacteria after PBS treatment)] × 100% [20]. The experiment was repeated three times, and the average results were calculated.

### 2.9. Statistical Analysis

All data were processed statistically and graphed using GraphPad Prism, version 7.00. We used one-way ANOVA, where *p*-values less than 0.05 were considered significant.

## 3. Results

### 3.1. Determination of Lactic Acid MIC Required to Develop NmC-BG Cell

No turbidity was observed visually after incubation for 24 h starting from volume 200 µL of lactic acid until the highest used volume. Moreover, no colonies of NmC bacteria were observed after spreading the BG pellets on BHI agar. This was consistent in the three performed replicates.

### 3.2. Identification Tests

#### 3.2.1. NmC-BG Evaluation by Scanning Electron Microscopy (SEM)

**Figure 1:**
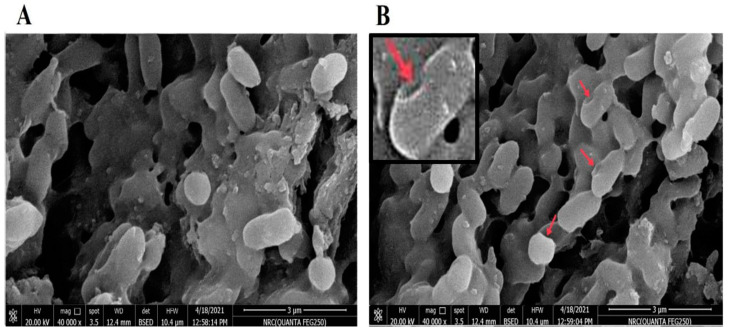
Scanning electron microscopy of treated and untreated *N. meningitidis* bacterial cells. A photo of scanning electron microscopy of NmC-BG cells (**B**) revealing the trans-membrane lysis tunnels in NmC-BG (small red arrows) compared to untreated bacteria (**A**).

The golden-coated NmC-BG and untreated bacteria were analyzed by SEM, showing that some cells of NmC-BG had the basic cell morphology of the bacteria but with some surface modification of the cell envelope compared to untreated bacteria. The formation of pores on the surface of BG cells indicated the formation of a trans-membrane lysis tunnel on the BG cell surface (Figure 1).

#### 3.2.2. Agglutination Test Using Pastorex™ Meningitis Kit

Agglutination was observed during 10 min with BG cells compared to negative polyvalent control latex, which remained negative, ensuring that the BG cells were *N. meningitidis* C (Figure S1).

### 3.3. Analysis of Protein

#### 3.3.1. Quantification of Released Proteins

The mean quantity of released cytoplasmic protein (µg/mL) of three experiments using a spectrophotometer, taking into consideration the protein content of un-inoculated media, was 492.29 µg/mL.

#### 3.3.2. Protein Gel Electrophoresis and Western Immunoblotting Analysis

##### Protein Gel Electrophoresis

**Figure 2:**
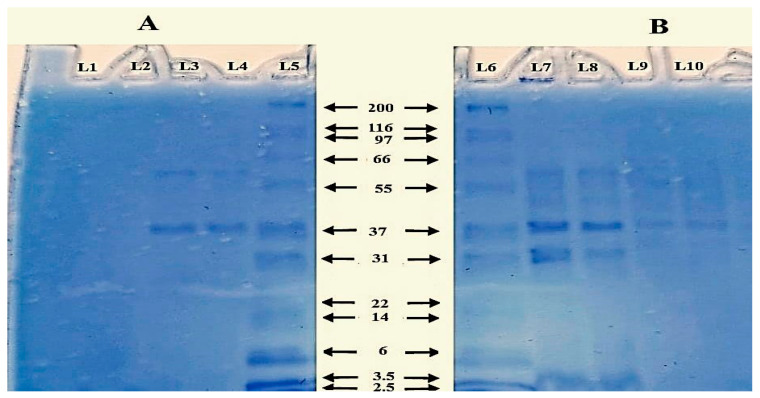
SDS-PAGE analysis of the protein content of treated and untreated NmC cells. A photo of 12% SDS-PAGE showing (**A**) the protein content of the supernatants of untreated bacterial culture (Lanes 1–2) and BG cell culture (Lanes 3–4) against a protein marker (Lane 5). (**B**) The protein content of untreated bacterial cell pellets (Lanes 7–8) and BG pellets (Lanes 9–10) against a protein marker (Lane 6).

The protein content of BG pellets Lanes (9–10) was much less than that of the untreated cells (Lanes 7–8) (Figure 2B). Another confirmation of the release of cytoplasmic protein to the supernatant was observed in bands (Lanes 3–4) that showed prominent protein bands compared to that of the supernatant of intact bacterial cells that showed almost no sign of protein (Lanes 1–2) (Figure 2A).

##### Western Immunoblotting Analysis

**Figure 3:**
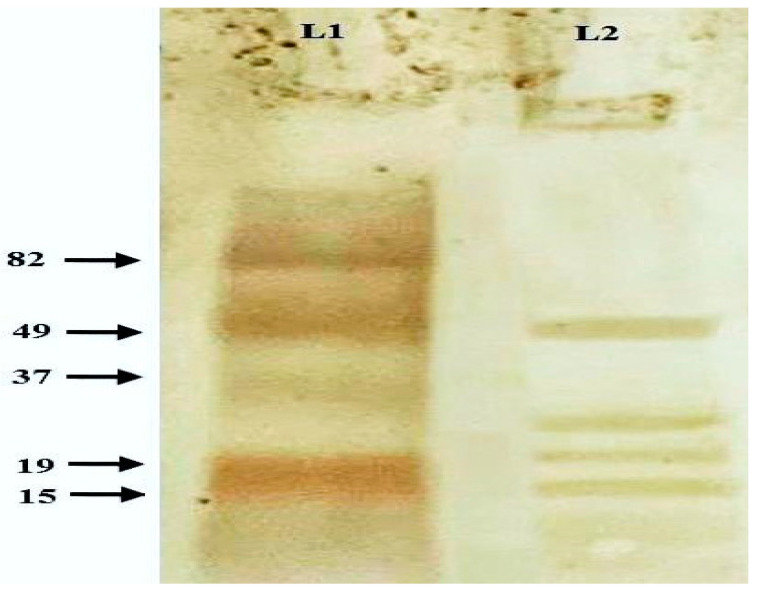
Western immunoblotting analysis of the protein cell wall of NmC-BG reacted animal BG-derived antibodies. The nitrocellulose membrane image showing the antigenic protein bands of BG pellets (Lane 2) against a pre-stained protein marker (Lane 1).

Sera from NmC-BG vaccinated animals were able to react with the cell wall protein antigens of *N. meningitidis* and displayed different protein bands at approximately 15, 19, 26, and 49 KDa (Figure 3).

### 3.4. Determination of DNA Content

#### 3.4.1. Quantification of DNA

The remaining DNA in the BG pellets was measured with an average value of 8.07 ng/µL compared to the untreated bacteria at 48.83 ng/µL. On the other side, the mean concentration of genomic DNA from the supernatant of BG was 40.27 ng/µL.

#### 3.4.2. DNA Gel Electrophoresis

**Figure 4:**
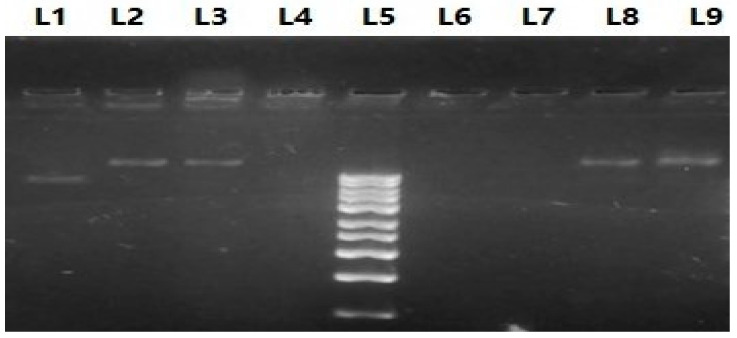
Gel electrophoresis of treated and untreated NmC cells. A photo of agarose gel showing the DNA contents of BG cells (Lanes 6, 7), untreated bacterial cells (Lanes 8, 9), and supernatant of BG culture (Lanes 1–3) against 1 Kbp Ladder (Lane 5), empty lane to separate the DNA ladder from the samples (Line 4).

Agarose gel electrophoresis was used to confirm the release of genomic DNA from the BG cells into the culture medium (Figure 4). The absence of DNA bands was observed in BG samples (Lanes 6, 7) when compared to the bacteria strain bands (Lanes 8, 9). Clear bands of the released DNA from BG cells to the supernatant were also observed (Lanes 1–3).

### 3.5. Safety Assessment

#### 3.5.1. Viability Test

No viable cells were observed on blood agar plates after incubation for three days at 37 °C in a 5% CO_2_ incubator (Thermo Fisher, USA) in the three performed replicates.

#### 3.5.2. Abnormal Toxicity Test

All animals survived until the end of the observation period. No change in their weight or general activity, and they showed no signs of illness.

#### 3.5.3. Bacterial Endotoxin Test

Gel formation was detected in the undiluted BG vaccine, while no endotoxin was detected in the whole dilutions. The obtained result after calculation was less than the sensitivity of LAL reagent × lowest dilution = 0.25 × 10 =2.5 EU/mL.

### 3.6. Assessment of Immune Response in Rats Sera

**Figure 5:**
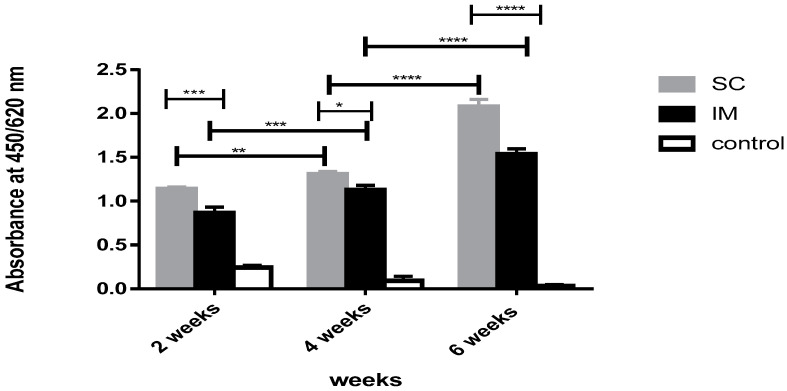
Immune response of SC route versus IM route after immunization with NmC-BG. A bar chart showing antibody response in rats measured in terms of absorbance values in weeks 2, 4, and 6 after immunization with BG vaccine against PBS (control) through subcutaneous (SC) and intramuscular (IM) immunization routes. Error bars represent the standard deviation from the mean. * Indicates significance with *p* < 0.05, ** indicates significance with *p* < 0.01, *** indicates significance with *p* < 0.001, and **** indicates significance with *p* < 0.0001.

Concerning BG vaccine preparation, the antibody response was elevated in the sera of the vaccinated rats when compared to that of non-vaccinated control rats (*p* < 0.0001). The IgG antibody response in the SC group showed a significantly higher response during the immunization periods (*p* < 0.01). The highest response was recorded in week 6 (*p* < 0.0001), as shown in (Figure 5). On serial dilution of the sera, the chosen dilution (4-fold) was significantly high, reaching up to a 32-fold increase after the third dose of immunization compared to the calculated cut-off of the control group.

On the other hand, the sera IgG response of the rats injected via the IM route showed an elevation during the immunization period (*p* < 0.001), as manifested in (Figure 5). On serial dilution of the sera, the chosen dilution (4 folded) was significantly high, reaching up to 24-fold after the third dose of immunization compared to the calculated cut-off of the control group.

Regarding the SC and IM groups, there was a significant difference between the obtained immune response of the SC group when compared to that of the IM group after each dose, with notable differences after both the first and third doses (*p* < 0.001), as illustrated in (Figure 5).

For the commercial vaccine (Meningococcal A&C, Walvax), the antibody response was elevated in the sera from the vaccinated group than that of the non-vaccinated control group (*p* < 0.0001).

**Figure 6:**
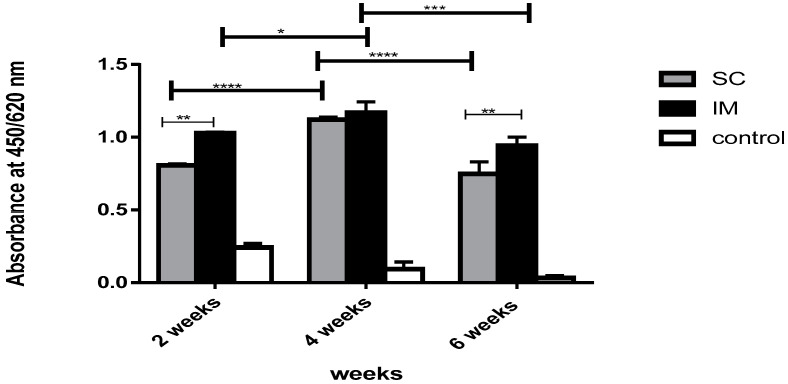
Immune response of SC route versus IM route after immunization with meningococcal (walvax) vaccine. A bar chart showing antibody response in rats measured in terms of absorbance values in weeks 2, 4, and 6 after immunization with the commercial meningococcal vaccine against PBS (control) through subcutaneous (SC) and intramuscular (IM) immunization routes. Error bars represent the standard deviation from the mean. * Indicates significance with *p* < 0.05, ** indicates significance with *p* < 0.01, *** indicates significance with *p* < 0.001, and **** indicates significance with *p* < 0.0001.

The immune response of the SC group showed an increase at week 2 and week 4 (*p* < 0.0001) with a significant decrease in week 6 (*p* < 0.0001), as shown in (Figure 6). The chosen dilution (4 folded) was significantly higher, reaching up to 12-fold after the third dose of immunization, than the calculated cut-off of the control group. While in the IM group, the immune response increased in week 2 and week 4 (*p* < 0.05) with a sudden decrease in week 6 (*p* < 0.001). The chosen dilution (4 folded) was significantly higher, reaching up to 15-fold after the third dose of immunization, than the calculated cut-off of the control group.

In the comparison between the SC and IM groups, there was a significant difference between the immune response of the IM group and that of the SC group after the first and third doses (*p* < 0.01); however, after the second dose, there was no significant difference between the two groups (*p* = 0.85).

**Figure 7:**
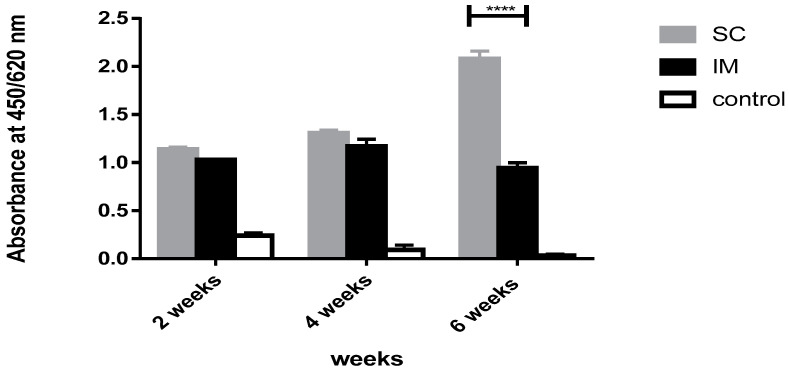
Immune response of SC route of BG vaccine versus IM route of the commercial vaccine. A bar chart showing antibody response in rats measured in terms of absorbance values in weeks 2, 4, and 6 after immunization with BG (SC) and commercial meningococcal vaccine (IM). Error bars represent the standard deviation from the mean. **** indicates significance with *p* < 0.0001.

Regarding the comparison between BG (SC route) and commercial vaccine (IM route), no significant difference between BG (SC route) and commercial vaccine (IM route) was observed at weeks 2 and 4 (*p* > 0.05). On the other side, after the third dose of the BG vaccine, the immune response of the BG vaccine drastically increased compared to that of the commercial vaccine (*p* < 0.0001) (Figure 7).

### 3.7. Bactericidal Activity

The functional antibody response of sera from vaccinated groups with the BG vaccine and the commercial vaccine was tested against virulent strains. Remarkably, rats vaccinated with the BG vaccine showed significantly higher bactericidal activity than the non-vaccinated control group (*p* < 0.0001).

#### 3.7.1. The Bactericidal Activity in Vaccinated Groups with NmC-BG

**Figure 8:**
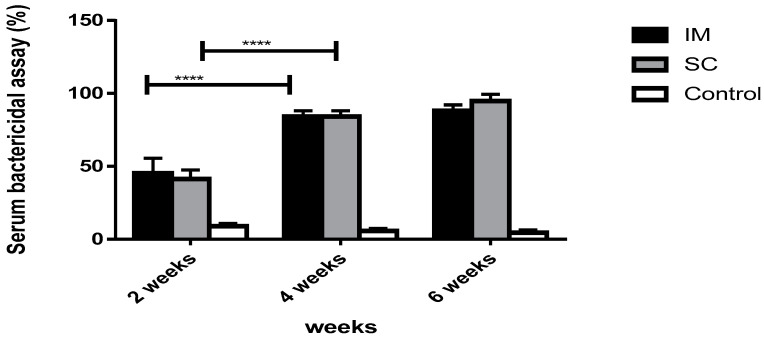
Serum bactericidal activity of rats vaccinated with NmC-BG after 2, 4, and 6 weeks. A bar chart showing the serum bactericidal activity of rats immunized with BG vaccine through intramuscular (IM) and subcutaneous (SC) routes against PBS (control). The bactericidal activity of the serum was determined after incubation with the *N. meningitidis* strain for 1 h. Error bars represent the standard deviation from the mean. **** indicates significance with *p* < 0.0001.

In vaccinated groups, the SBA increased significantly via SC and IM routes after the first dose (41.33% and 45.33%, respectively) and the second dose (84% and 84%, respectively). However, there was no significant difference in the SBA in both groups after the last vaccine dose (94.67% and 88%, respectively). Comparing both routes, there was no significant effect on the SBA percentage throughout the whole immunization period (*p* > 0.05), as shown in (Figure 8).

#### 3.7.2. The Bactericidal Activity in Vaccinated Groups with Commercial Vaccine

**Figure 9:**
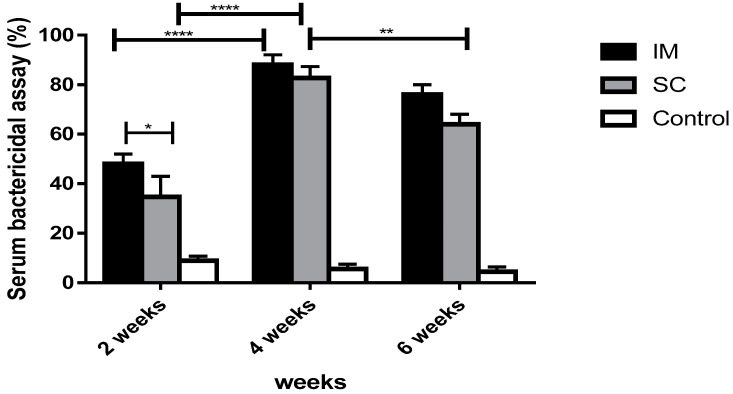
Serum bactericidal activity of rats vaccinated with a commercial meningococcal vaccine after 2, 4, and 6 weeks. A bar chart showing serum bactericidal activity of rats immunized with commercial vaccine through intramuscular and subcutaneous routes against PBS (control). The bactericidal activity of the serum was determined after incubation with the *N. meningitidis* strain for 1 h. Error bars represent the standard deviation from the mean. * Indicates significance with *p* < 0.05, ** indicates significance with *p* < 0.01, and **** indicates significance with *p* < 0.0001.

In immunized groups with the commercial vaccine, the bactericidal activity levels were significantly elevated after the first dose (IM: 48%, SC: 34.7%) and second dose (IM: 88%, SC: 82.7%), with a notable decrease after the third dose (IM: 76%, SC: 64%). The SBA of the intramuscularly vaccinated group was significantly higher than the subcutaneous group after the first dose (*p* = 0.0275), while they were almost similar in their SBAs after the second and third doses (*p* > 0.05) (Figure 9).

#### 3.7.3. The Bactericidal Activity in Vaccinated Groups NmC-BG versus Commercial Vaccine

**Figure 10:**
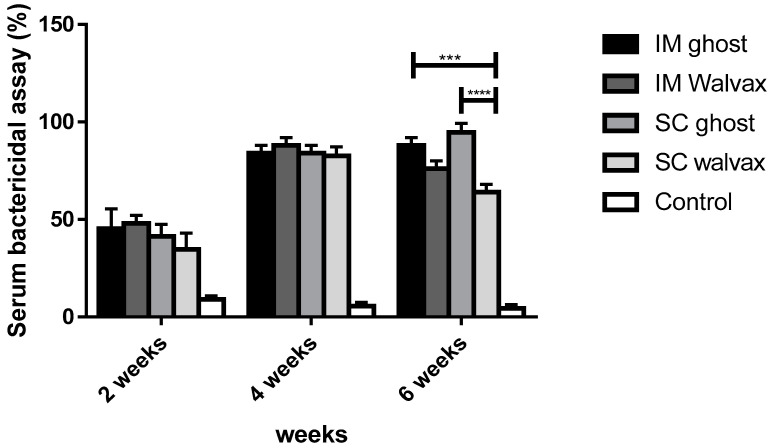
Serum bactericidal activity of rats vaccinated with NmC-BG and commercial meningococcal vaccine. A bar chart showing serum bactericidal activity in rats immunized with BG vaccine and commercial meningococcal vaccine through intramuscular and subcutaneous routes against PBS (control). The bactericidal activity of the serum was determined after incubation with the *N. meningitidis* strain for 1 h. Error bars represent the standard deviation from the mean. *** indicates significance with *p* < 0.001 and **** indicates significance with *p* < 0.0001.

There was no significant difference during the immunization period in BG and commercial vaccine groups via IM (*p* > 0.05), but in the case of the SC route, there was a significant difference between BG and commercial vaccine after the third dose (*p* < 0.0001). A remarkable difference was recorded between the BG vaccine via the IM route and the commercial vaccine SC route after the third immunization dose (*p* = 0.0002) as well (Figure 10).

## 4. Discussion

In our study, we tried a novel approach; the whole-cell vaccine through the production of NmC-BG, which provides an effective, economical, and potentially safe means of preventing diseases, and, moreover, it is T cell-dependent [27].

Several chemicals are claimed to inhibit bacterial cell growth. Chemical production of the BG vaccine by using MIC has been performed since 2013 [4]. This protocol was used for Gram-positive bacteria [25], Gram-negative bacteria [28], yeast vaccine [29], and viral BG vaccine [30]. Sodium hydroxide was used alone or in combination with other chemicals. A recent protocol for BGs production was introduced by Rabea et al. [6], in which tween 80 was used instead. It was able to evacuate Gram-negative *Salmonella enterica serovar typhimurium* from its cellular content [6].

In our study, the production of the NmC-BG vaccine was based on the MIC of lactic acid. This was carried out in approximately seven production steps [6] from the upstream process, bacterial cultivation to the downstream process, obtaining the BG. This is highly praised when compared to multi-step production, reaching up to 17 steps of high cost and longtime process for obtaining purified polysaccharide vaccine [11]. Many chemicals are used to remove proteins and nucleic acid, while in our study, we only used half-normal saline followed by a simple centrifugation step.

Lactic acid, used together with tween 80 in this protocol, lowered the pH of the overnight culture of the bacteria from 6.52 to 3.28, in which the BG was obtained. pH value was less than that used in Rabea et al. protocol [6]. This may be attributed to the fact that each strain has its own conditions for BG production, which have to be studied and modified.

SEM showed that the basic cell morphology of BGs was not affected compared to the wild-type *N. meningitidis* cells, but some cells had a little change in their outer membrane. This was similar to but less prominent than the effect of sodium hydroxide on *Vibrio parahaemolyticus* [31]. Trans-membrane tunnels were observed on the surface of BG cells. This was enough to evacuate the proteins and DNA contents.

For the main concept of BG production, almost empty cells were obtained. This was illustrated by the intense protein bands of the untreated bacterial pellets compared to that of the BG after analysis by protein gel electrophoresis. On the other hand, when we tested the diluted supernatants obtained from both treated and untreated bacteria suspensions, no protein bands were visualized in the SDS-PAGE of the supernatant of the untreated cell culture, unlike that of the treated cells that showed intense protein bands. This can be explained by the release of the cytoplasmic content to the outer medium. These results were additionally confirmed by spectrophotometry. Similar results were obtained by Hyun et al. in the production of *Vibrio parahaemolyticus* ghosts (VPGs) [31]. Comparable results were also obtained with the addition of sodium hydroxide to *Klebsiella pneumoniae* [28].

Also, the percentage of cytoplasmic DNA was measured by a NanoDrop spectrophotometer. Results showed that 82% of cytoplasmic DNA was released in the culture medium. This was emphasized by DNA gel electrophoresis, which demonstrated intense DNA bands in the culture supernatant samples. These findings resemble that of *Klebsiella pneumoniae* BG cells [28].

To investigate the safety of produced BG, a viability test was performed, and no viable cells were detected on blood agar. Safety was confirmed by the pharmacopeial abnormal toxicity test in addition to the bacterial endotoxin test. By performing the bacterial endotoxin test, it was less than 2.5 EU/mL. This may suggest that the lipopolysaccharide of NmC-BG was lost by lactic acid. This revealed that the NmC-BG vaccine was safe enough to be taken without causing a fever effect, and this may be due to using lactic acid as it was proven to have a liberating lipopolysaccharide effect [9].

The immunogenicity of the BG vaccine was investigated by ELISA technique, and the serum bactericidal test was compared to the available purified polysaccharide commercial vaccine (Meningococcal A&C, Walvax). NmC-BG and commercial vaccines are different in the manufacturing process as NmC-BG is a whole cell while the commercial vaccine is a purified polysaccharide. In our study, NmC-BG induced a significant immune response in immunized rats via all injected routes. This resembled the results obtained by *Salmonella enteritidis* after treatment with sodium hydroxide. Yet, in our study, the SC route was more effective than the IM route [20]. Moreover, the immunogenicity of meningococcal polysaccharides after three vaccination doses was much lower than that of NmC-BG.

Moreover, Western blotting analysis depicted the antigenic protein bands in the NmC-BG cell walls after reaction with sera from vaccinated animals. Comparable results were also obtained with *Salmonella typhimurium* BG [21].

While measuring the antibody titer as a suitable indicator for the induction of antibodies, the SBA test is considered the hallmark of immunity against meningitis. The serum bactericidal titer is defined as the reciprocal serum dilution causing ≥50% killing of the bacteria [32].

A serum bactericidal titer of ≥1:4 is considered a protective one when measured with human complement [33]. This was achieved in our study in rats’ sera with four-fold dilution. After mixing with bacteria, more than 50% were killed after the second and third doses, which was more than what was obtained in the previous study of the *Salmonella enteritidis* BG vaccine produced using sodium hydroxide [20]. Moreover, our NmC-BG vaccine excelled over the commercial vaccine in both antibody response and serum bactericidal effect after the third dose of immunization.

## 5. Conclusions

Our study demonstrated the production of dead and intact NmC-BG cells using tween 80 and lactic acid. Immunization with BG cells not only led to a significant humoral response but also an efficient SBA against the NmC strain. This paves the way to produce a promising, safe, protective, and cost-effective vaccine candidate against the NmC strain. However, more clinical studies are required to fully characterize this newly developed BG vaccine.

## Data Availability

Data will be available upon request.

## Supplementary Materials

The following supporting information can be downloaded at: https://www.mdpi.com/article/10.3390/vaccines11010037/s1, Figure S1: Agglutination test using Pastorex™ Meningitis kit; Figure S2: SDS-PAGE analysis of the protein content of treated and untreated NmC cells; Figure S3: Gel electrophoresis of treated and untreated NmC cells.
