# Supplementary material for: Production and Characterization of Bacterial Ghost Vaccine against Neisseria meningitidis
Source: Vaccines (Basel). 2022 Dec 23;11(1):37. doi: 10.3390/vaccines11010037 (PMC9865227; doi:10.3390/vaccines11010037)
Supplement: Supplementary file 1 [file vaccines-11-00037-s001.zip › vaccines-1986463-supplementary.pdf]

Production and characterization of bacterial ghost vaccine against *Neisseria meningitidis*

Randa H. Ali [1], Mohamed E. Ali [1] and Reham Samir [2,*]

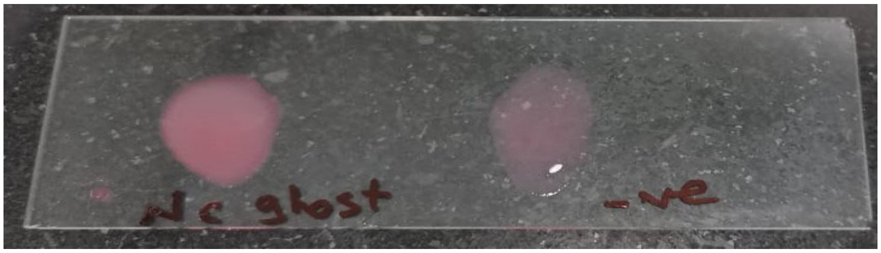

Figure S1. Agglutination test using Pastorex™ Meningitis kit.

A serological test showing positive agglutination reaction of BG cells with rabbit antibodies specific for N. meningitidis C compared to negative result obtained with negative polyvalent control latex.
